# Utilization of Syndromic Vaginitis Diagnostic Testing Reduces 6-Month Follow-Up Outpatient Service Healthcare Costs—A Real-World Data Analysis

**DOI:** 10.3390/healthcare12222204

**Published:** 2024-11-05

**Authors:** Azia Evans, Maren S. Fragala, Pallavi Upadhyay, Andrea French, Steven E. Goldberg, Jairus Reddy

**Affiliations:** HealthTrackRx, 1500 I-35 W, Denton TX 76207, USA; maren.fragala@healthtrackrx.com (M.S.F.); pallavi.upadhyay@healthtrackrx.com (P.U.); janie.french@healthtrackrx.com (A.F.); steven.goldberg@healthtrackrx.com (S.E.G.)

**Keywords:** health economics and outcomes research, healthcare resource utilization, molecular diagnostics, gynecology infections, polymerase chain reaction

## Abstract

Background/Objectives: Vaginitis is a common infection among women of reproductive age. Although various diagnostic methodologies exist, diagnosis without the utilization of available diagnostic tests remains prevalent. This study aimed to assess downstream healthcare utilization and the cost of patients with and without diagnostic testing. Methods: This retrospective, observational study utilized the IQVIA PharMetrics^®^ Plus database from July 2020 to October 2023. Patients with an index claim (ICD-10 code indicating vaginitis) were categorized into two cohorts: those who received a syndromic polymerase chain reaction (PCR) test and those who had no documented test on the index date or within two days. Total and service-specific healthcare resource utilization and costs were assessed for 6 months following the index event. This study was designed to inform how Syndromic Vaginitis PCR testing is used to make treatment decisions and to track outpatient and inpatient healthcare utilization for 6 months post index date represented by cost. Results: Patients who received a Syndromic Vaginitis PCR test had significantly fewer outpatient medical services in the 6 months following initial diagnosis compared to those who received no diagnostic test. This was largely attributed to a substantial decrease in other medical service visits, resulting in mean cost savings of USD 2067 (Syndromic PCR = USD 6675, SD = USD 17,187; No Test = USD 8742, SD = USD 29,894) (*p*-value 0.0009). Conclusions: Many vaginitis patients do not receive testing, but Syndromic Vaginitis PCR testing may be an effective diagnostic tool for reducing costs associated with vaginitis infections.

## 1. Introduction

Vaginitis is a highly prevalent condition that affects an estimated 10 million women in the U.S. annually and 75% of women in their lifetime [1,2]. Vaginitis can be caused by several distinct etiologies; however, symptoms remain consistent across the infectious causes. These include abnormal vaginal discharge, itching, burning odor and irritation, which typically present in women during their reproductive age (14–49 years of age) [3,4]. Bacterial vaginosis (BV) is the most common cause of vaginitis, accounting for up to 50% of all cases, followed by candidiasis and trichomoniasis, but over 30% of cases have unknown causes [2,3]. BV is caused by shifts in the normal vaginal microbiota and is associated with increased risk of human immunodeficiency virus, chlamydial and gonococcal infections [3,5]. Other infectious causes of vaginitis include *Mycoplasma genitalium* and herpes simplex virus in viral vaginitis cases [6,7,8].

The overall healthcare burden in North America for BV alone exceeds USD 1.2 billion annually and the direct costs double from poor outcomes attributable to vaginitis complications [9]. Given the overlap in symptoms between the different infectious causes of vaginitis, identifying the infectious agent(s) is key prior to initiating therapy, though empiric prescribing remains a prevalent practice [10]. Studies have shown that up to 50% of prescriptions for vaginitis given were inappropriate and patients receiving empiric therapy were more likely to return for follow-up visits within 90 days [10,11]. In addition, patients with vaginitis currently receive sub-optimal work-ups [12]. Further confounding empiric clinical diagnosis is the fact that chlamydial and gonococcal infections, the predominant infectious causes of vaginitis, can lead to similar symptoms but require different treatment regimens [13]. Standard, low-cost, in-office tests for common causes of vaginitis are available, consisting of vaginal pH measurement, “whiff” testing (production of a fishy odor when 10% potassium hydroxide is added to a slide containing vaginal fluid) and microscopic evaluation in patients presenting with milky vaginal discharge [14]. Due to the manual nature of these tests and physician variability, the sensitivity and specificity is widely variable [11]. A number of molecular-based polymerase chain reaction (PCR) tests, both FDA-approved and laboratory-developed, are available for vaginitis testing and yield higher sensitivity and specificity than the low-cost, in-office tests [15,16,17]. Syndromic approaches to vaginitis diagnostic testing allow for all causes of infectious vaginitis to be tested at once and have demonstrated improved diagnostic accuracy compared with clinical diagnosis [18]. Subsequently, the improved sensitivity and specificity of molecular tests could reduce the number of diagnostic tests that need to be ordered and decrease the need for follow-up visits in patients that might have otherwise been misdiagnosed. In addition, accurate diagnoses could minimize unnecessary prescriptions and help initiate timely and appropriate treatment. Since sexually transmitted infections can lead to pelvic inflammatory disease, early identification of infections can prevent the risk of complications and associated medical costs such as radiology.

PCR tests are on average more expensive, ranging from USD 35 to USD 240, than low-cost in-office tests, estimated at USD 15, and empiric prescribing; however, evidence is accumulating that use of PCR-based tests can drive down overall healthcare costs and improve patient outcomes [19]. Despite recent recommendations to incorporate PCR-based testing for vaginitis in practice guidelines, the healthcare outcomes and costs related to vaginitis utilizing advanced molecular syndromic-based diagnostics are largely unknown, as there have been limited healthcare economics and outcomes research (HEOR) studies to date [19].

The primary aim of this study was to assess the total outpatient utilization costs of patients who received a Syndromic Vaginitis PCR test compared to patients who had no diagnostic tests performed on or within two days of the date of service. The secondary aims include a breakdown of costs in outpatient medical services to determine the impact of Syndromic Vaginitis PCR testing on cost in different sectors of outpatient services. A tertiary endpoint was to assess the impact of Syndromic Vaginitis PCR testing on antibiotic prescribing rates. This overall study was designed to inform how Syndromic Vaginitis PCR testing is used to make treatment decisions and to track outpatient and inpatient healthcare utilization for 6 months post index date represented by cost.

## 2. Materials and Methods

### 2.1. Study Design

We conducted a retrospective cohort study using the IQVIA PharMetrics^®^ Plus claims database from 1 July 2020 to 31 October 2023 (study period). PharMetrics Plus is a health plan claims database comprising fully adjudicated medical and pharmacy claims for more than 210 million unique enrollees since 2006. This database is representative of the commercially insured U.S. national population for patients under 65 years of age. Related data can be accessed through publicly available all-payer claims databases offered by a limited number of states. As a retrospective study using secondary data, no interventions were made for patients during this study. In compliance with the Health Insurance Portability and Accountability Act (HIPAA), patient data included in the analyses were de-identified; therefore, this study was not subject to Institutional Review Board (IRB) review. Analysis of existing and anonymized data falls within the exempt criteria 45 CFR 46.101(b)(4) of HHS regulations for the protection of human subjects in research.

### 2.2. Cohort Selection

Adult women (aged ≥ 18 years) with ≥1 non-ancillary claims with a diagnosis for or a symptom suggestive of vaginitis, vulvovaginitis, dysuria, or other inflammation of the vagina from 1 January 2021 to 30 April 2023 were identified. The date of the first qualifying claim for vaginitis or related symptoms was considered the index date.

Patients included in the analysis were further required to have 6 months of continuous enrollment in health plans prior to and after the index date. The 6-month period prior to the index date was termed as the baseline period, and the 6-month period after the index date was termed the follow-up period. Patients with missing or invalid age or sex were excluded from the analysis.

**Figure 1:**
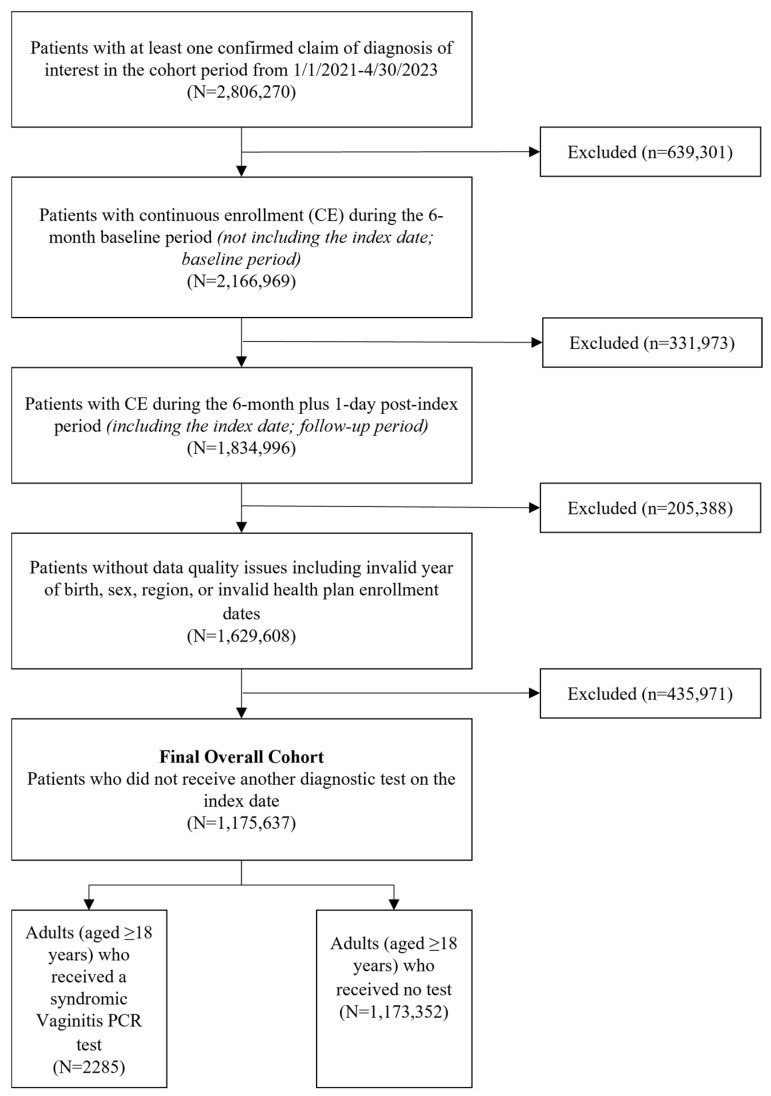
Attrition of patients with documented vaginitis symptoms. Patients enrolled in this study were required to have continuous enrollment for the 6 months before and after the index visit. Patients were excluded for data quality concerns and the final cohort determination was dictated by the diagnostic test or lack thereof associated with the index claim.

These patients were categorized depending on the diagnostic test administered on the index date, or lack thereof, into two mutually exclusive subcohorts: 1. patients who received a Syndromic Vaginitis PCR test (HealthTrackRx, Denton, TX, USA) on the index date were identified using a combination of Current Procedural Terminology (CPT)/Healthcare Common Procedure Coding System (HCPCS) codes for PCR tests and National Provider Identifier (NPI) codes for laboratories providing these specific tests (NPI: 1689639544, 1326743535, 1619346640, 1881352979, 1790470763) (Syndromic Vaginitis PCR cohort); and 2. patients who did not receive any diagnostic test of interest for vaginitis on the index date or within 2 days after (No Test cohort) (Figure 1).

### 2.3. Measures and Outcomes

Patient demographic characteristics such as age, sex, and geographic region (US Census region) of residence were assessed on the index date. Clinical comorbidities and the utilization of relevant treatments for vaginitis, as well as all-cause baseline total healthcare costs, were assessed during the 6-month baseline period. Charlson Comorbidity Index (CCI) score [20] (continuous and categorical) was assessed, along with chronic conditions such as cancers, congestive heart failure, hepatitis and renal failure. During the 6-month follow-up period, all-cause total, service-specific (overall outpatient, physician office visits, emergency room [ER] visits, other medical services [e.g., radiology, outpatient surgery, ancillary services and other service claims not classified in other outpatient subcategories]), inpatient and prescription medication healthcare resource utilization (HCRU), including the proportion of patients using the health resource and number of visits per patients, as well as associated healthcare costs, were assessed. These expenditures excluded the costs associated with the index event. In this study, the term costs represents the paid amounts.

### 2.4. Statistical Analysis

Baseline characteristics and outcomes for the Syndromic Vaginitis PCR and No Test cohorts were compared using appropriate statistical tests. For the continuous variables, a parametric *t*-test was used to compare the means and a Wilcoxon Rank-Sum test to compare the medians. For the categorical variables, Chi-Square tests were used to compare proportions and Fisher’s exact tests were used when more than 20% of categories had expected frequencies less than 5. A *p*-value of <0.05 was considered statistically significant.

## 3. Results

### 3.1. Patient Cohort Characteristics

Patient demographics were assessed for each cohort of this study and patients receiving the Syndromic Vaginitis PCR test (Mean = 38.4, SD = 18.2) were on average younger than those who received no diagnostic test (Mean = 42.7, SD = 18.6, *p* < 0.0001) (Table S1). In total, 37.2% of the Syndromic Vaginitis PCR cohort were between the ages of 18 and 34, while only 22.6% of the No Test cohort were in that same age bracket. Geographic distributions were comparable between the two groups, with both cohorts having most patients in the South and Midwest regions. However, this distribution was slightly shifted, with the Syndromic Vaginitis PCR cohort having a higher representation in the South and Midwest regions of the U.S., while the No Test cohort had slightly more patients in the Northeast and West regions (Table S1). While the adoption of PCR testing for vaginitis has increased in recent years, this analysis identified that a majority (1,173,351) of patients in this study had no diagnostic test associated with the initial index claim.

Clinical characteristics were also assessed prior to the index event, with a slightly higher proportion of patients in the Syndromic Vaginitis PCR cohort having a CCI of 0 (78.3%) compared to the No Test cohort (75.6%). The mean CCI for the Syndromic Vaginitis PCR cohort was 0.6, while the mean for the No Test cohort was 0.7 (*p* = 0.0584) (Table S2).

### 3.2. Healthcare Resource Utilization

**Figure 2:**
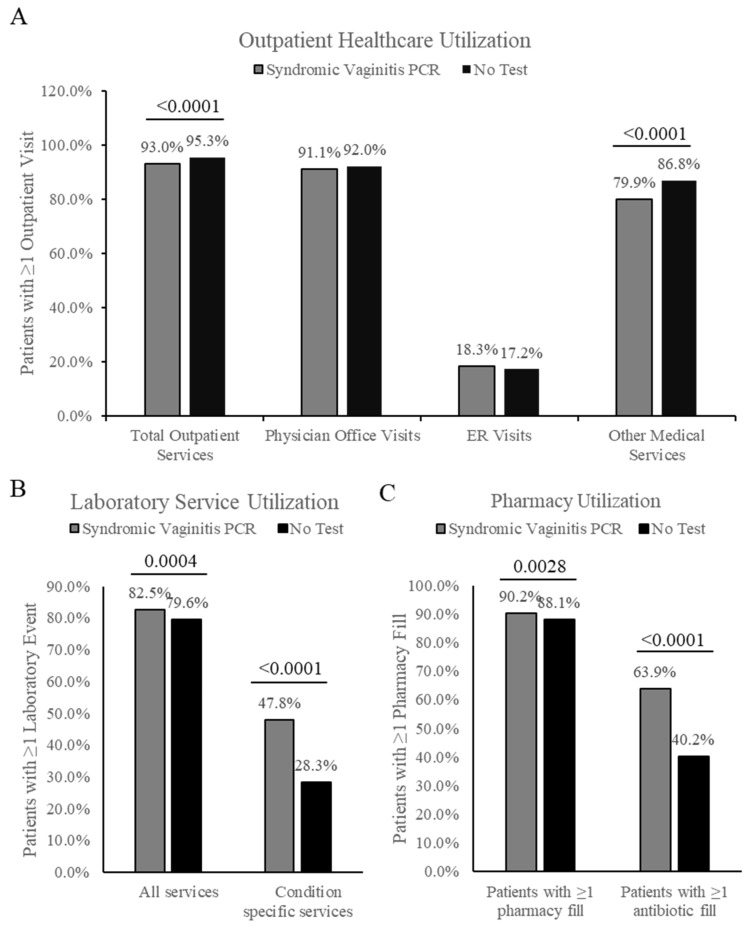
Healthcare utilization by service line. (**A**). Percent of patients with one or more claim during the 6-month follow-up period for outpatient services, including total outpatient services, physician office visits, ER visits, or other medical services including ancillary outpatient services. (**B**). Percent of patients with one or more claim associated with a laboratory service during the 6-month follow-up. Assessed as all services or services specific to vaginitis. (**C**). Percent of patients with one or more outpatient pharmacy fill during the follow-up period, divided into all pharmacy fills and pharmacy fills specific to antibiotic prescriptions.

Patients in the Syndromic Vaginitis PCR cohort had significantly fewer total outpatient medical services in the 6 months following the index date (mean = 18.1, SD = 32.1) compared to the No Test cohort (mean = 20.7, SD = 28.6) (*p* < 0.0001) (Table 1). This difference was largely driven by a reduction in other medical services, with patients in the Vaginitis PCR cohort averaging 9.9 other medical services (SD = 27.1) while the No Test cohort averaged 12.2 other medical services (SD = 20.8) (*p* < 0.0001) (Table 1). The Syndromic Vaginitis PCR cohort also had an as small but significantly lower proportion of patients receiving any outpatient service (93%) compared to the No Test cohort (95.3%) (*p* < 0.0001) (Figure 2A). Like the mean outpatient service findings, this difference was attributable to a reduction in other outpatient medical services (Syndromic Vaginitis cohort = 79.9%, No Test cohort = 86.8%) (*p* < 0.0001) (Figure 2A). No significant difference was observed in the number of physician office visits or emergency room (ER) visits, and the percentage of patients with greater than or equal to one visit in both categories was consistent.

Differences in laboratory and pharmacy services were also assessed. A small but significant difference was found in the utilization of all laboratory services between the two cohorts, with 82.5% of the Syndromic Vaginitis PCR cohort receiving one or more service, while only 79.6% of the No Test cohort received one or more laboratory service during the 6-month follow-up period (*p*-value = 0.0004) (Figure 2B). The difference in lab service utilization was greater when examining condition-specific services: 47.8% compared to 28.3% for the Syndromic Vaginitis PCR and No Test cohorts, respectively (*p*-value < 0.0001) (Figure 2B). The mean number of condition-specific laboratory services was also higher for the Syndromic Vaginitis PCR group (mean = 1.9, SD = 3.7) compared to the No Test group (mean = 0.7, SD = 1.6) (*p* < 0.0001) (Table 1). No significant difference was found in the mean number of pharmacy fills per patient during the 6-month follow-up period (Table 1). However, a small but significant increase was found in the percent of patients that had greater than or equal to one pharmacy fill (Syndromic Vaginitis PCR = 90.2%, No Test = 88.1%, *p*-value = 0.0028) (Figure 2C). When examined further, the difference in pharmacy utilization may be attributed to a higher proportion of patients in the Syndromic Vaginitis PCR cohort receiving one or more antibiotic fill (Syndromic Vaginitis PCR = 63.9%, No Test = 40.2%, *p*-value < 0.0001) (Figure 2C).

### 3.3. Healthcare Cost

Differences in healthcare costs were assessed between the two cohorts and the Syndromic Vaginitis PCR cohort was found to have significantly lower outpatient medical costs compared to the No Test cohort, resulting in a mean cost difference of USD 1568 (*p*-value < 0.0001) (Table 2). Mean costs were not significantly different when examining the costs attributed to physician office visits or ER visits. The largest difference in mean cost was attributed to other medical services, which encompassed radiology, outpatient surgery, ancillary services, and other service claims not otherwise captured within the other outpatient subcategories. Patients receiving a Syndromic Vaginitis PCR test had mean other medical service costs of USD 2206 (SD = USD 8895), while the costs in the No Test cohort were USD 3640 (SD = USD 13,743, *p*-value < 0.0001) (Table 2). No significant difference was found between the cohorts for inpatient cost. Outpatient pharmacy spend was also assessed, and while there was no difference in total pharmacy spend between the two cohorts, patients in the Syndromic Vaginitis PCR group had a higher cost from antibiotic prescriptions specifically (Syndromic Vaginitis PCR = USD 25, No Test = USD 15, *p*-value = 0.0314) (Table 2).

Total all-cause healthcare costs were also assessed for the two cohorts. Patients receiving the Syndromic Vaginitis PCR test had a mean all-cause healthcare cost of USD 6675 (SD = USD 17,187), while those patients who received no test had a mean cost of USD 8742 (SD = USD 29,894) (*p* < 0.0001) (Table 3), resulting in a mean allcause healthcare cost difference of USD 2068 per patient.

## 4. Discussion

Leveraging healthcare claims data, this study aimed to assess the real-world impact of multiplex syndromic PCR diagnostics for female patients suffering from vaginitis on 6-month follow-up healthcare utilization and costs compared to clinical diagnosis alone. Findings from this analysis suggest that the use of syndrome-driven PCR testing for vaginitis can result in a significant reduction in follow-up healthcare services and potential cost savings.

While several diagnostic solutions exist for the identification of vaginal infections, empiric diagnosis or diagnosis based on clinical symptoms alone continues to be widespread. Of the 1,175,637 patients with a diagnosis or symptoms of vaginitis that met the enrollment requirements for this study, only 2285 (0.2%) had a syndromic vaginitis PCR test performed. While this study did not aim to assess the impact of other diagnostic methodologies or include PCR testing performed by other companies, the differences in cohort size indicate that empiric diagnosis continues to be a dominant practice. This is often due to the rationale that symptoms are clinically distinct between the specific vaginitis etiologies; however, evidence suggests that as many as 42% of patients with vaginitis receive inaccurate treatment in the outpatient setting [11,21]. This is further compounded by the high rate of recurrence of these infections, with over 20% of patients seeking follow-up care within three months of their initial diagnosis [11,22].

In this study, we found that compared to patients receiving no diagnostic test, patients who received a syndromic PCR test had significantly fewer outpatient medical services in the 6-month follow-up period (Syndromic Vaginitis PCR = 18.1, No Test = 20.7, *p*-value < 0.0001). The reduction in both total outpatient services and other outpatient medical services corresponded to a significant reduction in healthcare costs in each category. The total difference in mean outpatient service costs was USD 1568 per patient, with most of that cost reduction attributed to other medical services and physician office visits. While physician office visits were not found to be significantly different in the Syndromic Vaginitis PCR cohort, a small reduction in utilization (Syndromic Vaginitis PCR = 7.9, No Test = 8.3, *p*-value = 0.1153) and subsequent costs (Syndromic Vaginitis PCR = USD 896, No Test = USD 1015, *p*-value = 0.1092) was still observed. The largest difference in cost was observed in the other medical services category (Syndromic Vaginitis PCR = USD 2206, No Test = USD 3640, *p*-value < 0.0001), which encompasses outpatient radiology, outpatient surgical services, and other ancillary services/procedures.

While patients receiving a syndromic vaginitis PCR test had fewer follow-up outpatient claims, patients in this cohort had higher utilization of laboratory and pharmacy services specific to antibiotic prescriptions. Despite this observed increase in select service utilization associated with syndromic PCR use, all-cause total healthcare costs were reduced by USD 2068 (*p*-value = 0.0009). Together, these findings may suggest that the observed laboratory service increase contributed to more effective treatment for these patients, ultimately leading to a reduction in follow-up care.

The large sample size of the group receiving no test is a highlight of this study, as it provides a comprehensive look at the costs associated with diagnosis based on clinical presentation alone. This study also benefits from the assessment of a diagnostic test only offered by a single laboratory, as this reduces variability resulting from different turnaround times, tested microorganisms, and assay performance. However, due to the analysis of a single diagnostic test for vaginitis, the cohort size receiving the Syndromic Vaginitis PCR test was smaller than the No Test group. This study also evaluated macro changes in costs without further evaluation of an individual or single category.

Findings from this analysis should be interpreted within the context of a few limitations. Retrospective claims analysis is limited by data utility and incomplete data, as data were collected prior to the study aims being set. Additionally, it is possible that observations were attributable to factors other than the use of the test, being a real-world analysis. This study did not analyze provider characteristics associated with ordering the test. Further studies would benefit from this analysis to better understand test ordering and utilization patterns. Despite the limitations, this real-world analysis offers important findings to the literature on population-level management of vaginitis by evaluating the long-term impact of a newer syndromic test in a large patient population. Additional studies leveraging medical record data are needed to draw further conclusions on the frequency of repeat office visits, impact on provider treatment decision-making, and specific services that syndromic vaginitis PCR testing may impact in matched cohorts.

## 5. Conclusions

The utilization of a syndromic PCR test for vaginitis led to reduced total healthcare costs, primarily attributed to a reduction in medical services costs. Healthcare providers may consider utilizing syndromic PCR testing for patients with vaginitis as a way to ensure accurate diagnosis and reduce unnecessary follow-up medical services for their patients.

## Figures and Tables

**Table 1:** Outpatient medical service utilization per patient. Mean outpatient medical services for patients enrolled in each cohort assessed as total outpatient services or specific service lines.

Total Outpatient Medical Services	Mean	SD	Median	IQR	*p*-Value
Syndromic Vaginitis PCR	18.1	32.1	10	18	
No Test	20.7	28.6	12	21	<0.0001
*Outpatient pharmacy fills*					
Syndromic Vaginitis PCR	13.9	20.3	8	14	
No Test	13.2	18.9	7	15	0.0737
*Physician office visits*					
Syndromic Vaginitis PCR	7.9	10.2	4	8	
No Test	8.3	11.8	5	8	0.1153
*ER visits*					
*Number of ER visits (among all patients)*					
Syndromic Vaginitis PCR	0.3	1.1	0	0	
No Test	0.3	1.0	0	0	0.5553
*Number of ER visits (among patient with ≥1 ER)*					
Syndromic Vaginitis PCR	1.7	2.0	1	1	
No Test	1.7	2.0	1	1	0.7098
*Other medical services*					
Syndromic Vaginitis PCR	9.9	27.1	4	10	
No Test	12.2	20.8	6	13	<0.0001

**Table 2:** Post-index healthcare costs per patient. Mean all-cause healthcare costs assessed per cohort during the 6-month follow-up period.

Cost Measures	Mean	SD	Median	IQR	*p*-Value
**Total Outpatient Medical Services**					
Syndromic Vaginitis PCR	USD 3460	USD 9606	USD 1047	USD 2622	
No Test	USD 5028	USD 15,114	USD 1420	USD 3888	
Total Outpatient Cost Savings	USD 1568				<0.0001
*Physician office visits*					
Syndromic Vaginitis PCR	USD 896	USD 1324	USD 481	USD 876	
No Test	USD 1015	USD 3559	USD 511	USD 914	0.1092
*ER visit*					
Syndromic Vaginitis PCR	USD 358	USD 1544	USD 0	USD 0	
No Test	USD 373	USD 1749	USD 0	USD 0	0.6897
*Other medical services*					
Syndromic Vaginitis PCR	USD 2206	USD 8895	USD 266	USD 1243	
No Test	USD 3640	USD 13,743	USD 519	USD 2277	<0.0001
**Outpatient Pharmacy**					
*Total outpatient pharmacy costs*					
Syndromic Vaginitis PCR	USD 2023	USD 8619	USD 186	USD 719	
No Test	USD 2086	USD 11,245	USD 184	USD 760	0.7886
*Total costs specific to antibiotic prescriptions*					
Syndromic Vaginitis PCR	USD 25	USD 106	USD 6	USD 24	
No Test	USD 15	USD 221	USD 0	USD 8	0.0314
**Inpatient**					
Syndromic Vaginitis PCR	USD 1192	USD 8252	USD 0	USD 0	
No Test	USD 1629	USD 19,031	USD 0	USD 0	0.2726

**Table 3:** Total all-cause post-index healthcare costs. Mean all-cause total healthcare costs assessed per cohort during the 6-month follow-up period.

Total Healthcare Costs	Mean	SD	Median	IQR	*p*-Value
Syndromic Vaginitis PCR	USD 6675	USD 17,187	USD 1609	USD 4682	
No Test	USD 8742	USD 29,894	USD 2145	USD 6270	
Total Healthcare Cost Savings	USD 2068				0.0009

## Data Availability

The data presented in this study are available through the IQVIA PharMetrics^®^ Plus claims database and include claims from 1 July 2020 to 31 October 2023.

## Supplementary Materials

The following supporting information can be downloaded at: https://www.mdpi.com/article/10.3390/healthcare12222204/s1, Table S1: Demographic characteristics during 6-month baseline period; Table S2: Clinical characteristics during 6-month baseline period; Table S3: Inpatient healthcare utilization over 6-month follow-up period; Table S4: Laboratory healthcare utilization over 6-month follow-up period.
