# Supplementary material for: Utilization of Syndromic Vaginitis Diagnostic Testing Reduces 6-Month Follow-Up Outpatient Service Healthcare Costs—A Real-World Data Analysis
Source: Healthcare (Basel). 2024 Nov 5;12(22):2204. doi: 10.3390/healthcare12222204 (PMC11593449; doi:10.3390/healthcare12222204)
Supplement: Supplementary file 1 [file healthcare-12-02204-s001.zip › healthcare-3249546-supplementary.pdf]

Supplemental Material

Table S1: Demographic Characteristics During 6-month Baseline Period

Baseline Demographic Characteristics	Vaginitis Syndromic PCR		No Test		p-value
**Age at study index date**					
Mean	38.4		42.7		<0.0001
SD	18.2		18.6		
Median	37		45		
**Age group** (n, %)					
<18	206	9.0%	116,695	9.9%	<0.0001
18-24	410	17.9%	102,027	8.7%	
25-34	442	19.3%	162,913	13.9%	
35-44	382	16.7%	203,037	17.3%	
45-54	356	15.6%	237,046	20.2%	
55-64	315	13.8%	251,889	21.5%	
65-74	106	4.6%	61,901	5.3%	
≥75	68	3.0%	37,844	3.2%	
**Geographic region**: (n, %)					
Northeast	26	1.1%	178,493	15.2%	<0.0001
Midwest	731	32.0%	298,064	25.4%	
South	1,286	56.3%	522,306	44.5%	
West	242	10.6%	174,489	14.9%	

Table S2: Clinical Characteristics During 6-month Baseline Period

Baseline Clinical Characteristics	Syndromic Vaginitis PCR		No Test		p-value
**Charlson Comorbidity Index (CCI), CDMF adaptation**					
Mean	0.6		0.7		0.0584
SD	1.6		1.7		
Median	0		0		
**CCI categories** (n,%)					
0	1,790	78.3%	886,830	75.6%	0.0105
1	240	10.5%	144,415	12.3%	
2	84	3.7%	52,066	4.4%	
3	40	1.8%	19,291	1.6%	
4	20	0.9%	7,349	0.6%	

5+	111	4.9%	63,401	5.4%

Table S3: Inpatient Healthcare Utilization Over 6-month Follow-up Period

Total Inpatient Utilization Per Patient	Mean	SD	Median	IQR	P-Value
**Average length of stay (among all patients)**					
Syndromic Vaginitis PCR	0.3	1.5	0	0	0.3
No Test	0.3	2.0	0	0	0.3430
**Average length of stay (among patient with ≥1 inpatient stay)**					
Syndromic Vaginitis PCR	5.9	4.8	4	4	
No Test	5.8	7.0	4	3	0.9149
**Number of inpatient stays (among all patients)**					
Syndromic Vaginitis PCR	0.1	0.4	0	0	
No Test	0.1	0.4	0	0	0.2083
**Number of inpatient stays (among patient with ≥1 inpatient stay)**					
Syndromic Vaginitis PCR	1.4	1.0	1	0	
No Test	1.4	1.0	1	0	0.9449
**Number of hospitalization days per patient (among all patients)**					
Syndromic Vaginitis PCR	0.4	3.8	0	0	
No Test	0.5	4.4	0	0	0.4361
**Number of hospitalization days per patient (among patient with ≥1 inpatient stay)**					
Syndromic Vaginitis PCR	9.6	15.8	4	7	
No Test	9.6	17.3	4	5	0.9978

Table S4: Laboratory Healthcare Utilization over 6-month Follow-up Period

Laboratory/Pathology Utilization	Mean	SD	Median	IQR	P-Value
**Laboratory/pathology (For all laboratory/pathology services)**					
Syndromic Vaginitis PCR	12.5	16.1	8	15	
No Test	9.8	15.2	5	12	<.0001
**Laboratory/pathology (For condition-specific tests)**					
Syndromic Vaginitis PCR	1.9	3.7	0	2	
No Test	0.7	1.6	0	1	<.0001
